# The Effects of Cryotherapy on Early Rehabilitation Following Total Knee Arthroplasty: A Prospective Cohort Study

**DOI:** 10.7759/cureus.50279

**Published:** 2023-12-10

**Authors:** Hamza Duffaydar, Huan Dong, Maha Jebur, Ejaz Mughal

**Affiliations:** 1 Surgery, Royal Wolverhampton NHS Trust, Wolverhampton, GBR; 2 Trauma and Orthopaedics, Royal Orthopaedic Hospital, Birmingham, GBR; 3 Trauma and Orthopaedics, Royal Wolverhampton NHS Trust, Wolverhampton, GBR

**Keywords:** length of stay, analgesia, pain, total knee arthroplasty, arthroplasty, cryotherapy

## Abstract

Purpose

The objective of this study was to investigate whether cryotherapy is effective in reducing pain, opioid consumption, and length of stay (LOS) in hospital following total knee arthroplasty (TKA).

Methods

This prospective cohort study included 191 consecutive patients who underwent TKA without having access to cryotherapy, followed by 193 consecutive patients who underwent TKA and received automated cryotherapy as part of the recovery phase. All patients had their surgical procedures performed by the same surgeons and received post-operative care by the same nursing, medical, and physiotherapy team. The pain score using the visual analog scale (VAS)and the amount of opioid used on the first three post-operative days were recorded along with the length of hospital stay.

Results

There was no difference in baseline characteristics between the two groups. The use of cryotherapy was associated with a reduced pain score on all three days compared to when cryotherapy was not used: Day 1 pain score was 5.2 versus 6.1 (p < 0.01), Day 2 was 3.6 versus 4.8 (p = 0.03), and Day 3 was 2.8 versus 3.8 (p < 0.01). Cryotherapy was also associated with a significant reduction in analgesia consumption on all three days. The median amount of Oramorph used on all three days in the cryotherapy group was 15.0 mg compared to 40.0 mg in the control arm (p < 0.01). Additionally, the LOS was shorter in the cryotherapy group, with a mean of 3.86 days versus 4.20 days in the control group (p = 0.02).

Conclusion

The use of cryotherapy following TKA was associated with decreased pain and opioid consumption along with a reduced time to hospital discharge compared to when no cryotherapy was used.

## Introduction

Total knee arthroplasty (TKA) is a well-established procedure for managing end-stage osteoarthritis, providing significant relief from pain and improved joint function [1]. Over 80,000 procedures are performed annually in the United Kingdom, and the demand keeps growing given the aging population [2,3]. Immediate post-operative pain can be associated with substantial pain, discomfort, and challenges in achieving optimal rehabilitation outcomes [4,5]. Effective pain management and reducing hospital length of stay (LOS) are crucial objectives in enhancing the overall quality of care and patient satisfaction in TKA [5,6]. Cryotherapy, or the application of cold therapy, has gained increasing attention as a potential adjunctive intervention in orthopedic post-operative care [7]. Cryotherapy is believed to have several beneficial effects, including reducing pain, inflammation, and edema while potentially expediting the recovery process [8,9]. Despite the growing interest in cryotherapy, there is limited empirical evidence regarding its specific impact on post-operative outcomes following TKA, particularly with respect to pain, opioid requirements, and LOS [10]. This prospective cohort study aimed to address this knowledge gap by investigating the potential associations between the use of cryotherapy in the immediate post-operative period following TKA on post-operative pain, analgesia utilization, and LOS. The primary outcomes of this study were the pain score on the visual analog scale (VAS) and opioid utilization following TKA. The hypothesis was that cryotherapy positively affects post-operative pain following TKA and is thus associated with reduced opiate usage in the recovery phase and a shorter LOS.

## Materials and methods

Patient selection

**Figure 1:**
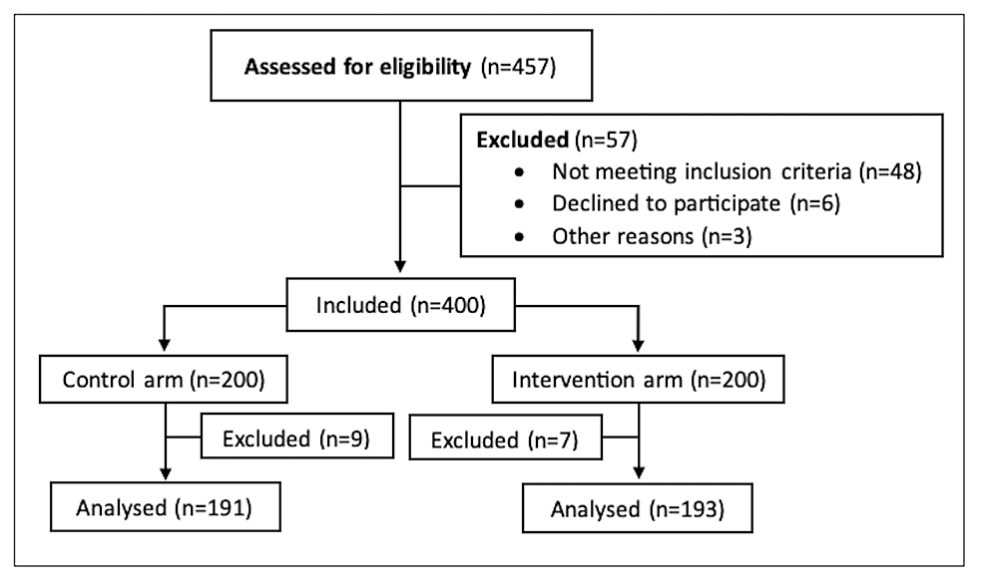
Flowchart showing inclusion criteria.

This prospective cohort study was conducted at an elective orthopedic teaching hospital between March 2022 and February 2023. The hospital review board reviewed the study and advised that further assessment by the institutional board for ethical approval was not required. Patients aged 18 to 80 years undergoing primary, unilateral TKA were invited to participate in the study, and written consent was obtained. The exclusion criteria included patients undergoing unicompartmental, bilateral, or revision knee arthroplasties. Of the 457 patients identified, 400 met the inclusion criteria. The use of automated cryotherapy at our hospital was introduced in September 2022. Patients undergoing TKA from March to August 2022 were assigned to the control group, while those operated on from September 2022 to February 2023 were assigned to the intervention arm, receiving cryotherapy routinely post-operatively. An additional seven and nine patients were excluded from the cryotherapy and control groups due to poor compliance in filling out pain questionnaires. In total, data from 193 patients in the cryotherapy group and 191 patients in the control group were analyzed. Figure 1 is a flow chart further illustrating the inclusion criteria.

Surgical technique

All patients received a primary unilateral TKA by experienced surgeons under general anaesthesia. At induction, they received 1g of tranexamic acid along with 1.5 g of cefuroxime as surgical prophylaxis. As per the surgeons’ approach, a pneumatic tourniquet was applied but not inflated unless they had trouble in achieving hemostasis. All knees were approached using a midline anterior incision with a medial parapatellar approach. The implant system used was the Stryker Triathlon Total Knee System®, which uses both cruciate-retaining (CR) and posterior-stabilized (PS) implants. Before skin closure, hemostasis was controlled using diathermy, and no wound drainage was used post-operatively. Patients received a further 500 mg of tranexamic acid six hours post-operatively, along with two further 750 mg doses of cefuroxime eight hours apart. Post-surgery, patients were also provided with two weeks of venous thromboembolism prophylaxis and were encouraged to commence early full weight-bearing activities under the guidance of a physiotherapist starting from the first day.

Intervention

Following TKA, patients in the intervention arm started receiving cryotherapy through an automated device. The device used was the Zamar Therapy Cube®, which allows a fixed temperature to be reached. The devices were set at 10 degrees Celsius, and patients started receiving cryotherapy within one hour of returning to the ward. The nursing staff applied the cryotherapy cuffs, and participants received four sessions daily, each lasting up to two hours. Participants in the control arm did not receive cryotherapy or any cold treatment.

Post-operative inpatient care

Following their return from the recovery unit, all patients were prescribed regular analgesia per the WHO analgesic ladder [11]. They received up to 4g of paracetamol and 240 mg of codeine phosphate per day, which was given regularly by the nursing team. They also had morphine prescribed for breakthrough pain delivered on an as-required basis. Patients could request 5-10 mg of Oramorph every hour with a maximum daily dose of 60 mg. Given the use of the electronic prescribing system at our hospital, the amount of Oramorph given to a patient could be monitored, and data could be recorded for analysis. Patients in the cryotherapy group received cryotherapy within one hour of returning to the ward and received a single session of two hours on the same day following their surgery. They then received four sessions every day, starting from Day 1 until the day of discharge, with each session lasting two hours. Patients in the control group received no cold treatment in the post-operative phase. All patients were reminded daily to complete a questionnaire with the VAS on a scale of 1-10 to rate the pain experienced from days 1 to 3, with a morning and afternoon score [12]. As part of post-operative recovery, all patients received up to one hour of physiotherapy daily starting from Day 1. The same team performed rehabilitation in both groups. Patients were discharged once they had achieved adequate pain control, knee flexion to at least 90 degrees, could mobilize independently with the aid of crutches, and completed a stairs assessment to satisfactory standards.

Data collection

Data collected included patient demographics such as gender, age, and ethnicity. Information such as BMI, American Society of Anaesthesiologists (ASA) grade [13], and previous TKA were obtained prospectively from the anesthetic pre-operative charts. For each patient, the admission and discharge date was recorded and used to calculate LOS. From the pain questionnaire, the VAS pain score for each patient daily was recorded for morning and afternoon sessions, and the mean was calculated for each day. The amount of Oramorph each patient received daily was recorded for the first three days, and this information was available on the electronic prescribing system. Data collection was undertaken by two independent surgical trainees (Hamza Duffaydar and Maha Jebur), and disparities were resolved by consensus.

Statistical analysis

A sample size calculation was performed to recruit enough patients to detect a minimum pain score improvement of 25%. This proportion was agreed upon by consensus among the authors as significant enough to impact the population and warrant implementation in hospitals. With a 95% confidence interval and a margin of error of 0.05, a sample size of at least 289 patients was required. Following the recommendations of the senior author (Ejaz Mughal), this number was increased to 400 patients to account for potential non-compliance, technical issues with the cryotherapy devices, or patients rescinding their consent later. When comparing baseline characteristics and outcome measures between the two groups, an unpaired t-test was used for data that followed a normal distribution. For data not normally distributed, the Wilcoxon-Mann-Whitney test was used to determine the significance of differences between the two groups. Continuous variables that were normally distributed were expressed as mean and SD, whereas those not normally distributed were displayed as median and interquartile range (IQR). Categorical variables were presented as the number and percentage of patients. A p-value of less than 0.05 was considered statistically significant, and statistical analysis was performed using IBM® SPSS® Statistics software version 21.0 (IBM Corp., Armonk, NY, USA).

## Results

**Figure 2:**
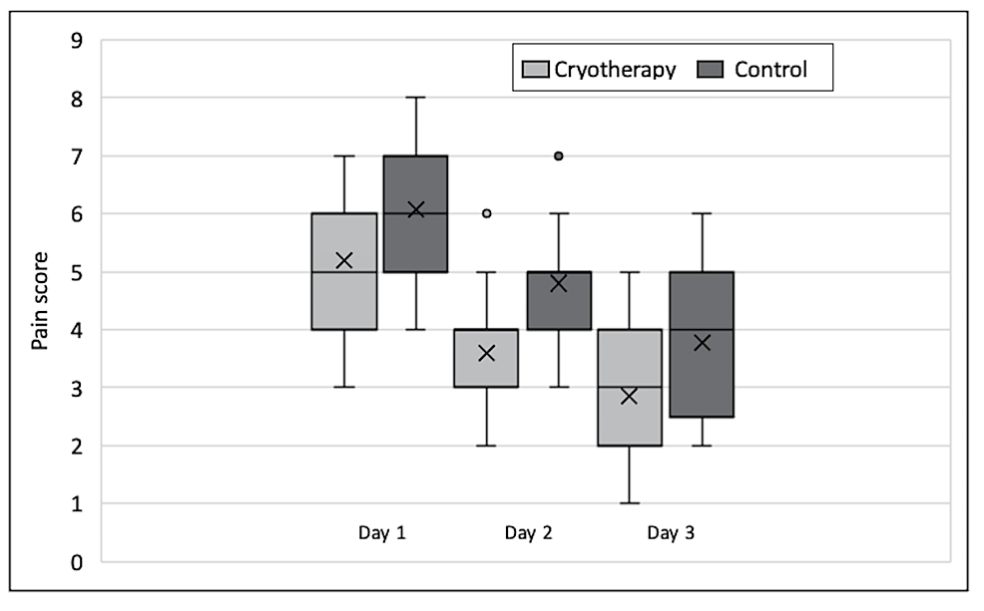
Boxplot showing pain score as measured using the visual analog scale (VAS) for both groups on each individual day.

There was no statistical difference in baseline characteristics between patients receiving cryotherapy and those in the control group, implying that both groups' populations were homogeneous (Table 1). The study outcomes are shown in Table 2. Patients receiving cryotherapy had reduced pain scores in all three days following TKA compared to those in the control arm. This difference was statistically significant throughout (unpaired t-test), and the greatest difference was observed on day 2 where the mean VAS score was lower by 1.2 in the cryotherapy group compared to the control arm. In both groups, there was a reduction in mean VAS score progressing from Day 1 to Day 3. Figure 2 further illustrates the difference in VAS scores between the two groups on all three days.

**Table 1:** Patient baseline characteristics. †Chi-squared test    *Unpaired t-test ASA: American Society of Anaesthesiologists; TKA: Total knee replacement. p-value <0.05 was considered a statistically significant difference between the two groups.

Outcome variable	Cryotherapy (n=193)	Control (n=191)	P-value
Gender: n(%)			0.253^†^
Male	82 (42.5)	69 (36.1)	
Female	111 (57.5)	122 (63.9)	
Age,years: mean (SD)	71.5 (8.9)	70.9 (8.3)	0.515^*^
BMI,kg/m2: mean(SD)	30.9 (5.3)	30.6 (5.7)	0.615^*^
Ethnicity			0.644^†^
White British, n(%)	159 (82.4)	148 (77.5)	
Asian	26 (13.5)	30 (15.7)	
Mixed	5 (2.6)	7 (3.7)	
Other	3 (1.6)	6 (3.1)	
ASA grade, n(%)			0.328^†^
I	47 (24.4)	42 (22.0)	
II	132 (68.4)	136 (71.2)	
III	14 (7.3)	13 (6.8)	
IV	0 (0.0)	0 (0.0)	
TKA contralateral knee: n(%)			0.201^†^
Yes	56 (29.0)	68 (35.6)	
No	137 (71.0)	123 (64.4)	

**Table 2:** Study outcomes comparing pain score, Oramorph usage, and length of stay between the two groups. *Unpaired t-test   †Mann-Whitney U test VAS: Visual analog scale; IQR: Interquartile range. Data are presented as mean (SD) or median (IQR) for both groups.

Outcome	Cryotherapy (n=193)	Control (n=191)	p-value
Mean pain score (VAS) - Day 1	5.2 (1.1)	6.1 (1.1)	< 0.01^*^
Mean pain score (VAS) - Day 2	3.6 (1.1)	4.8 (1.2)	0.03^*^
Mean pain score (VAS) - Day 3	2.8 (1.2)	3.8 (1.3)	< 0.01^*^
Median Oramorph (mg) - Day 1	10.0 (IQR 5.0 to 10.0)	25.0 (IQR 16.25 to 25.0)	0.04^†^
Median Oramorph (mg) - Day 2	5.0 (IQR 0.0 to 7.5)	10.0 (IQR 7.5 to 15.0)	< 0.01^†^
Median Oramorph (mg) - Day 3	0.0 (IQR 0.0 to 5.0)	5.0 (IQR 2.5 to 10.0)	< 0.01^†^
Median Oramorph (mg) - Total of three days	15.0 (IQR 5.0 to 22.5)	40.0 (IQR 27.5 to 50.0)	<0.01^†^
Mean time to discharge (days)	3.86 (1.1)	4.20 (1.5)	0.02^*^

**Figure 3:**
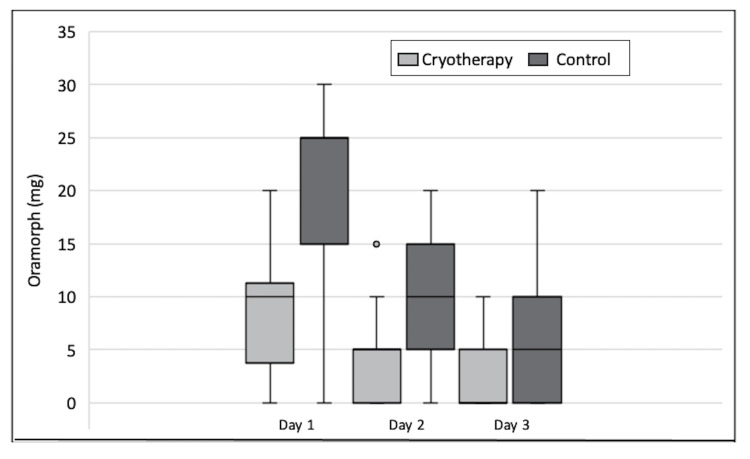
Boxplot showing breakdown of Oramorph usage per day for both groups.

**Figure 4:**
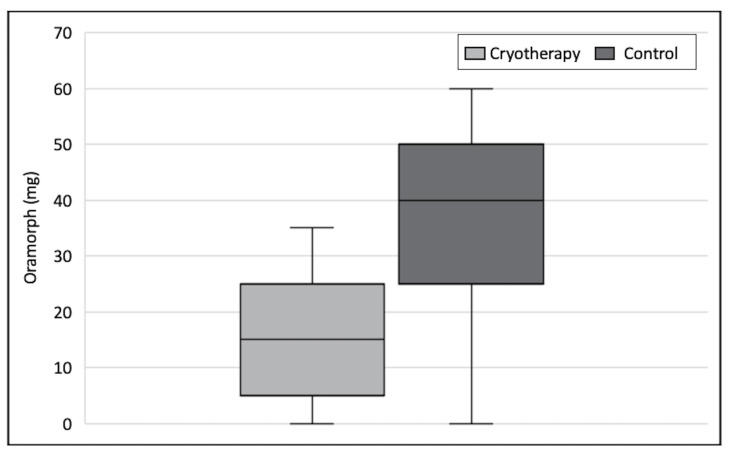
Boxplot showing total Oramorph usage in first three days for the two groups.

Patients receiving cryotherapy also used significantly less Oramorph compared to those in the control arm, and again, the difference was statistically significant on all three days (Mann-Whitney U test). Figure 3 shows the outcome of opiate use for each post-operative day in the form of a boxplot. The greatest difference was seen on Day 1 when the median amount of Oramorph used was 10.0 mg for those receiving cryotherapy as compared to 25.0 mg for patients in the control arm (p=0.04). The total amount of opiate used by patients on the first three post-operative days was also significantly lower (p<0.01). The median total amount of Oramorph was 15.0 mg in those who received cryotherapy compared to 40.0 mg in the control group. This represents a 2.7-fold reduction in opioid usage when cryotherapy was introduced. Figure 4 illustrates the total amount of Oramorph used by patients in the first three days for both groups.

**Figure 5:**
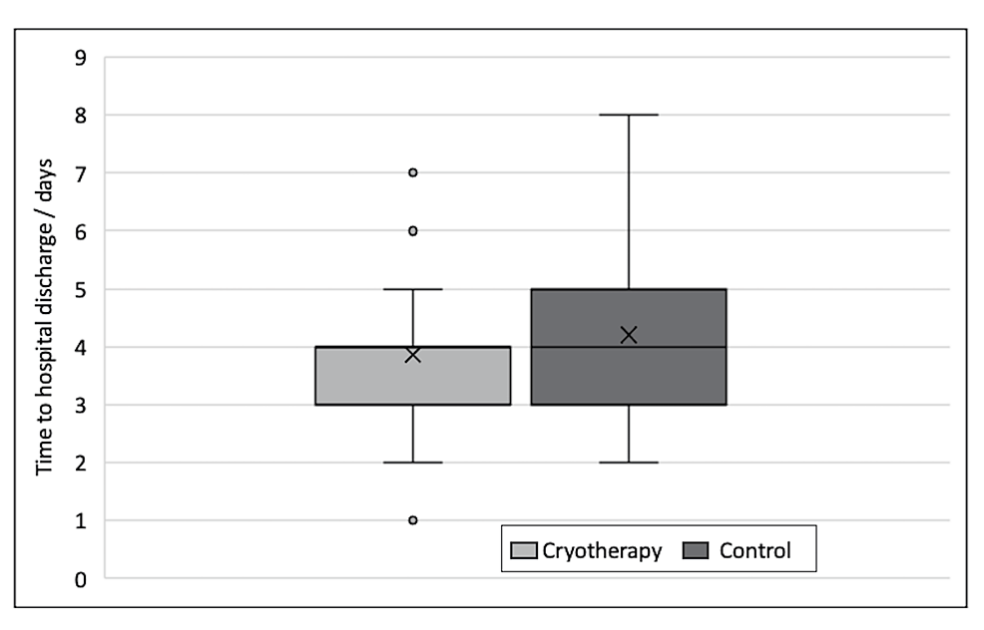
Boxplot showing length of stay (days) between the two groups.

The use of cryotherapy was associated with reduced LOS following TKA. The mean LOS was 3.86 days in patients receiving cryotherapy compared to 4.20 days for those in the control group. This difference of 0.34 days was statistically significant (p=0.02). Figure 5 is a boxplot comparing LOS in the two groups.

## Discussion

In this prospective cohort study, the baseline characteristics between patients in both groups were the same. The same surgeons performed patient operations and received peri-operative care from the same anesthetic team. During the post-operative recovery phase, the same team of nurses, medical doctors, and physiotherapists were involved in their care. The use of cryotherapy was associated with reduced post-operative pain, decreased analgesic requirements, and a decreased LOS compared to when cryotherapy was not used. Our findings suggest that implementing cryotherapy as part of the post-operative recovery phase would promote early functional recovery and reduce time spent in the hospital following TKA. When analyzing data from the National Joint Registry of England and Wales, pain following TKA was found to be the strongest predictor of patient dissatisfaction and reduced functional outcome, including the Oxford Knee Score [14]. Regression analysis also showed post-operative pain as the most important prognostic indicator for long-term dissatisfaction following TKA [15]. Our study showed a significant reduction in post-operative pain on all three days when cryotherapy was used. Given the findings of our study, we hope that patient satisfaction and functional outcomes will also be improved. A Cochrane review found low-level evidence that cryotherapy improves pain at 48 hours when measured using the VAS. They concluded that further robust studies are needed to evaluate the effect of cryotherapy on pain [16]. This was partly due to the small sample size of studies and the high risk of bias. Our study provides more robust evidence given our large sample size and, to the authors' knowledge, remains the largest study carried out to date, looking at the effects of cryotherapy on pain, usage of analgesia, and LOS.

The use of high amounts of opiate following TKA is thought to have a negative influence on post-operative rehabilitation. This is due to side effects such as nausea and dizziness, which may prolong a hospital stay and contribute to poor patient experience [10,17]. Our study shows that by using cryotherapy to reduce pain, opioid usage can be minimized. There was a 2.7-fold reduction in the total amount of opioids used during the first three days when cryotherapy was used. This is consistent with the results obtained in randomized controlled trials by Thijs E et al., which showed a 2.6-fold reduction, and Brouwers HF et al., which showed a 2-fold reduction in opioid usage when cryotherapy was used [18,19]. However, in both instances, the randomized controlled trials looked at Tramadol and Oxycodone usage, respectively. In our study, Oramorph was used instead. Nonetheless, the results may be comparable, given that they are all opioid-based medications. By introducing cryotherapy as part of standard recovery following TKA, the dependence on opioid usage and its harmful effects is minimized, leading to improved patient satisfaction. Cryotherapy was also associated with a significant reduction in LOS following TKA, a mean reduction of 0.34 days between the two groups. This difference was also observed in a prospective study by Kullenberg B et al., who reported a mean reduction of 1.4 days [20]. The estimated cost of an inpatient stay in our hospital was around £460/day, and the cost of each cryotherapy device was £55. When performing a cost analysis, an estimated £101.4 could be saved for every patient undergoing TKA if cryotherapy is used. This amount saved would be in the form of reduced LOS. Furthermore, with growing pressures on the NHS for orthopedic operations, any reduction in the length of hospital stay would be beneficial. In December 2020, it was estimated that around 13,000 patients had been waiting longer than a year for a hip or knee arthroplasty [21]. Those estimated figures were before the COVID-19 pandemic, which has since caused a significant rise in those numbers. As of March 2022, the British Orthopaedic Association reported a new record of 730,000 patients on the trauma and orthopedics waiting list [22]. Cryotherapy can potentially reduce waiting time for an arthroplasty while being more cost-effective for the hospital by reducing the LOS.

When interpreting the results of our study, several limitations need to be considered. Firstly, this study did not evaluate long-term clinical outcomes. We only looked at pain score and analgesic usage within the first three post-operative days when it was assumed the pain would be at its highest. Nor did we follow-up patients on their discharge to assess for any post-operative complications that might develop in the community. Secondly, patients and observers reporting outcomes of interest could not be blinded as data was collected prospectively when patients had the cryotherapy device on. Thirdly, we did not consider the pre-operative grading of arthritis and radiological outcomes when assessing baseline characteristics of patients. This has the potential to influence a patient's tolerance of pain and thus have a direct effect on the outcomes measured. Despite the limitations, this study remains the largest one to date, assessing the effects of cryotherapy on pain, analgesic usage, and time to discharge. Cryotherapy was associated with reduced post-operative pain, decreased analgesia requirements, and a reduced LOS. Given the findings of our study, we would encourage the use of cryotherapy as part of standard recovery following TKA.

## Conclusions

The use of cryotherapy following TKA is associated with reduced post-operative pain, reduced analgesia usage, and early functional recovery. Establishing its usage should be incorporated in post-operative recovery following knee arthroplasty.
